# A Pragmatic Approach to Resolving Technological Unfairness: the Case of Nike’s Vaporfly and Alphafly Running Footwear

**DOI:** 10.1186/s40798-020-00250-1

**Published:** 2020-05-24

**Authors:** Bryce Dyer

**Affiliations:** Department of Design & Engineering, Bournemouth University, Poole, UK

**Keywords:** Fairness, Technology, Running, Footwear

## Abstract

**Background:**

Technology is often introduced into sport to facilitate it or to improve human performance within it. On occasion, some forms of novel technology require regulation or prevention entirely to ensure that a sport remains fair and accessible. Recently, the Nike Vaporfly and Alphafly shoes have received some concerns over their appropriateness for use in competitive distance running.

**Methods:**

This paper evaluates the use of these shoes against an existing framework for sports technology discourse and adopts a pragmatic approach to attempt to resolve them.

**Results:**

It is proposed that the three concerns regarding cost, access and coercion cannot be ruled out but likely remain short-term issues. As a result, it is proposed that these running shoes are acceptable forms of technology but that ongoing vigilance will be required as such technologies develop further in the future.

**Conclusions:**

The Nike Vaporfly/Alphafly shoes do push the perceived acceptability of running shoes to the limits of the current sports regulations. However, the alleged gains have not manifested themselves to a level that could be considered excessive when reviewing historical performances or when evaluated against a set of well-cited criteria. The sport will need to adopt a stance of ongoing vigilance as such technologies continue to develop or be optimised in the future.

## Keypoints

- The use of the Nike Vaporfly/Alphafly running shoes does not generate a historically unusual level of performance improvement when evaluating the world record improvements and when using a performance improvement index.
- When using a peer-reviewed 10-point framework regarding sports technology acceptability, not all of its criterion was supportive of the shoes’ continued acceptability.
- The employed footwear technology is likely not yet optimised so further performance gains in the future may be possible.

## Introduction

In October 2019, Kenyan Eliud Kipchoge became the first runner to complete the 26.2-mile marathon running distance in under 2 h [1]. This attempt was known as the Ineos 1:59 Challenge. The Ineos project attempt was not recognised as a legal world record due to the event conditions it was held under and the nutritional, aerodynamic and pacing assistance that Kipchoge received. Notably, Kipchoge was wearing a pair of prototype Nike running shoes that were similar to the Nike ‘Vaporfly Next%’ shoes that he had when competing before. Likewise, during the same month, Brigid Kosgei broke the women’s world record in the marathon by completing the distance in 2:14:04. She achieved this record when wearing a pair of the Vaporfly NEXT% [2].

A previous study has attempted to predict the probable limits of the men’s marathon record [3]. However, since then, the maximum projections in that study have already been exceeded by official records by circa 2 min and nearly by 4 if the illegitimate Ineos project was eligible to be included. Additionally, in 2018, runners wearing the Nike Vaporfly 4% running shoe broke world records in the 100-km, marathon, half marathon and 15-km running distances [2]. These increases could be seen as unusual when it is considered; it has been proposed that sports performance will eventually indicate a reducing or plateaued nature in their rate of improvement [3]. To counter this, it has been proposed that any significant gains in performance sport in the future will be as a result of technical or technological innovation [4]. This implementation of new sports technology has seen a significant impact in *cycling*, the *100-m sprint* and the *javelin* [5] as well as the *pole vault* [5], *long jump*, *high jump*, *triple jump* [4], *amputee sprinting* [6] and *swimming* [7]. As a result, the innovation, design and application of technology used in competitive sport are of paramount importance to athletes looking to optimise their best possible performance in the future. However, there have been some recent concerns that the design of Nike’s Alphafly or Vaporfly shoes could be considered too advantageous or could be considered unfair [2]. There have also been several circumstances in peer-reviewed literature whereby technology specifically relating to feet or footwear has also been proposed as being unfair. These have included the use of prosthetic limbs for running [8], the use of prosthetic limbs for long jumping [9], speed skating footwear [10] and the use of the ‘brush shoe’ track spike design that was manufactured by Puma and ultimately banned [11].

The concerns directed towards Nike’s shoes were proactively attempted to be addressed recently with a proposal to regulate footwear designs with a series of arbitrary, yet quantifiable design constraints [2]. However, this proposal inferred that the shoes were already inappropriate for use without considering the wide range of philosophical issues that have been reported in such discussions before [12], coupled with the broader debate that such controversy can require [6].

The discourse surrounding whether sports technology is controversial or fair is well documented and was summarised by Dyer [13]. This discourse is based on the premise that sports technology requires an ethical foundation rather than an attitude to win at all costs [14]. The use of the word ‘foundation’ by Freeman implies that a philosophical understanding is required prior to then obtaining a scientifically robust outcome. As a result, whilst Burns and Tam’s approach could be considered pragmatic by attempting to provide a practical solution to shoe regulation [2], it could be considered premature until a broader debate on their acceptability had taken place.

The Dyer study resulted in a systematic review of 31 reported cases of sports technology controversy, and this led to an 11-item summary of factors that affected sports technology inclusion [13]. This summary is reproduced in Table 1.

**Table 1:** Summary of sports technology impact

Criterion	
Harm or health (to the athlete or others)	
Unnaturalness	
Unfair advantage	
Coercion	
Safety and spectator appeal	
Integrity of the game, harm to or advantage over the sport itself, or the ‘spirit of the sport’	
Deskilling and reskilling	
Dehumanisation	
Cost (or excess cost)	
The internal goods of a sport	
Equal opportunity or access	

In the past, the issues surrounding the ‘internal goods of a sport’ have been discussed synonymously with the integrity of a sport [15]. As a result, this list could be reduced instead to ten separate themes. The issues that surround using criteria like those in Table 1 is that such debates can be highly philosophical and can provide a lack of actionable outcomes. For example, whilst some studies debate the philosophical acceptability of sports technology [12, 16], such research can sometimes adopt what would be seen as a utopian stance [17]. Such a stance can lack the means to resolve the problem in reality. However, a pragmatic approach may offer a viable means to address such shortcomings. The philosophical origins of pragmatism originate from ancient Greece and means ‘action’ [18] and were later conceived by modern philosophers such as Charles Pierce to be a philosophy of science with the logic of enquiry at its centre [19]. Pragmatists typically opt for methods and theories that are more useful to humanity in addressing problems in specific contexts [20] or using a problematic situation as a starting point of inquiry [19]. The implementation of a pragmatic approach is particularly pertinent to the Nike shoe case as a governing body may be expected to determine if the use of such technology is in the sports’ best interests in the future. This situation occurred in the Speedo full-length swimsuit [21] and prosthetic limb use in able-bodied sport [22] case studies. This paper will aim to adopt a pragmatic stance and explore the potential legality of the Nike Vaporfly/Alphafly running shoes.

## Materials and Methods

The potential legality of the Nike Vaporfly/Alphafly running shoes was explored by modifying and reframing the items listed in Table 1 as questions. The questions intentionally adopted a pragmatic approach with their lines of inquiry as recommended by Bartle and Shields [19] so that the discussion provided outcomes that would then potentially be suitable for sports stakeholders to act upon. These questions were addressed by utilising the existing literature. The structure of this paper utilised a similar structure to previous studies that have explored the legality of technological issues in sport [23].

The adaptation of the pragmatically reframed research questions in Table 1 were as follows:

Are the Nike Vaporfly/Alphafly shoes harmful to the health of the athlete using them?

Are the Nike Vaporfly/Alphafly shoes unnatural?

Do the Nike Vaporfly/Alphafly shoes provide an unfair advantage?

Could the introduction of the Nike Vaporfly/Alphafly shoes coerce athletes to want to use them?

Does the use of the Nike Vaporfly/Alphafly shoes contribute towards spectator appeal?

Does the use of the Nike Vaporfly/Alphafly shoes affect the integrity of the sport or provide an advantage over the sport itself?

Does use of the Nike Vaporfly/Alphafly shoes deskill or reskill the sport?

Does the use of the Nike Vaporfly/Alphafly shoes somehow dehumanise the sport?

Could financial cost be a barrier to using the Nike Vaporfly/Alphafly shoes?

Are the Nike Vaporfly/Alphafly shoes inaccessible to some athletes?

## Results and Discussion

### Are the Nike Vaporfly/Alphafly Shoes Harmful to the Health of the Athlete Using Them?

The criterion of *harm* relates to the result of an injury or any temporary or permanent damage inflicted on an athletes’ health either directly or via side effects [24]. In the summary provided by Dyer [13], most historical cases of harmful sports technology centres on the use of performance enhancing drugs or chemically based ergogenic practises. However, it is conceivable that use of a new form of sports equipment or technology could cause more harm than those it seeks to replace.

Running shoes in general have been a source of debate regarding their influence on running-based injuries either positively [25] or negatively [26]. However, in the negative cases, the cause is often not the shoe in isolation but moreso the shoe causing injuries through negative changes to the runner’s biomechanics [26]. None of the published studies that have evaluated the shoe technologies (like those adopted in the Nike Vaporfly or Alphafly shoes) have noted any concerns regarding injuries over that of the other shoes they were tested against to date. In fact, it was actually noted by Candau et al. [27] that any muscle damage to the runner might actually be ameliorated by using extremely compliant running shoes such as those evident in the Nike Vaporfly designs [28]. Whilst it is conceded that it has been proposed that running barefoot could reduce injuries from those wearing shoes [29], this view has also been challenged in other studies. Even if this were the case, this would only then suggest that *all* footwear should be banned, not just the Nike Vaporflys. In light of the fact that running footwear is already in service and that no issue can be attributed to the Nike footwear specifically, it is argued that there is no evidence that the Vaporfly/Alphafly shoes are currently endangering the user [30].

### Are the Nike Vaporfly/Alphafly Shoes Unnatural?

Naturalness has been proposed as an entity that tampers with the body and interferes with nature with the overarching outcome being a performance that could not be achieved otherwise [24]. Likewise, dehumanisation has been suggested as being a consequence of disenchantment with technological progress [31]. The definition of whether a form of technology is ‘unnatural’ to its user or not could be argued to be fundamentally flawed when the very nature of manufactured products is that they are man-made and unnatural entities to begin with. On this basis alone, all forms of man-made technology such as footwear would have to be banned. Shoes used for distance running already act in an unnatural manner as they manipulate the biomechanical patterns of runners and increase shock absorption that the runner would not be able to obtain without them [32]. Whilst the concept of unnaturalness has been levied against performance-enhancing drugs due to their non-essential need [12], it would be arguably unrealistic to also apply this concern to running shoes when they have been used virtually universally for over 100 years. Pragmatically, footwear of some description is ultimately required to run competitively or that, without it, the nature of distance running itself would be changed.

### Do the Nike Vaporfly/Alphafly Shoes Provide an Unfair Advantage?

The Nike Zoom Vaporfly design has been formally assessed against several conventional designs of running shoes [33]. The design of this footwear is unique in that it combines an innovative highly compliant and resilient midsole material with a stiff embedded composite plate [33]. In this case, it was designated as a ‘prototype’ design and the results suggested that this feature reduced the energetic cost of running by 4% on average and may have been even greater if the normalisation for shoe weight had not taken place [33]. The study theorised that the shoes’ advantages likely originated from their elastic properties and subsequent energy return. This performance increase was then estimated to lead to a potential improvement in running velocity at marathon world record pace of circa 3.4% [32] or more recently 2.64% [34]—depending on the shoe and the runner involved. A recent investigation [35] was built upon the findings of the 2018 Hoogkamer et al. study by suggesting that a metabolic saving was due to superior energy storage in the midsole foam, a lever effect originating from the carbon-fibre plate acting on the ankle joint mechanics and a stiffening effect of this plate on the MTP joint [36]. However, any proposed gains may not always be of exactly the same reported magnitude when applied to other types of runners or running velocities. However, it was suggested that even prior to the Ineos project, these shoes would likely provide enough performance enhancement to help break the 2-h barrier for the marathon distance [35]. Ultimately, it is not contested that the design of the shoes offers an advantage, but the previous studies that support this did not determine whether this magnitude could be construed as ‘unfair’. Therefore, a pragmatic response to this concern would be necessary to quantify not only what advantage exists but also how this compares to a relative and reasonable baseline in performance. The measurement of technological progression has been attempted before to indicate such changes [5]. The performance improvement index (PII) provided a means of relative comparison via a mathematically calculated score [5]. This index was developed to demonstrate unusual leaps in performance that could then retrospectively be attributed to technological change. The PII primarily assesses changes in sports performances over a period of time such as their speed, distance or event completion duration, and this can then form a relative comparison to other sports that rely on different metrics. The PII has also been used to explore the impact of World Wars 1 and 2 upon world records [7] and on the impact of technological innovation in Olympic jumping events [4]. The PII cannot currently isolate the exact proportion of impact of sports technological change, but it is a complementary tool to help inform debate.

When considering timed events over fixed distances, Haake defines the PII [4] as:

$$ \mathrm{Index}=\left[{\left(\frac{t^1}{t^2}\right)}^2-1\ \right] $$ × 100

This formula is a linear regression of work done by a body overcoming aerodynamic drag when moving through air with a fixed density. It comprises a first timed performance *t*_1_ divided by a second performance *t*_2_. The rest of the formula converts the change between two performances and then expresses this as a percentage. This formula assumes events requiring motion to be dominated by aerodynamics. However, it should be noted that a key limitation when comparing the PII from different sporting disciplines is that air resistance increases exponentially as velocity increases [37]. For example, the contribution of air resistance is approximately 8% of the total energy for a 5000-m runner but approximately double that for a 100-m sprinter [38]. As a result, the magnitude of the PII index may be slightly skewed when comparing events that result in different absolute and average speeds. Additionally, it could also be argued that it would have been more appropriate in principle for this formula to be based on the *power* to overcome aerodynamic drag rather than the *work done*. However, it could be assumed that this was not pursued by Haake due to such data not being readily available from runners as it would have been from other sports like cycling.

In the case of distance running, the Nike Alphafly/Vaporfly shoes have been used in both the marathon and half marathon running distances [2]. The ratified and legitimate world records for these distances are published by the athletics governing body, World Athletics [39]. Whilst the model of shoe worn by athletes for world records since 2016 cannot always be verified, the record to record PII increases can be calculated. These results are summarised in Table 2.

**Table 2:** Summary performance improvement index changes

	PII positive change range	PII largest change since 2016	World record PII change ranking
Marathon: men’s	0–7.2	2.1	14th/38
Half marathon: men’s	0–7.5	0.98	11th/32
Marathon: women’s	0.1–9.2	2.0	21st/30
Half marathon: women’s	0–6.2	0.7	15th/27

It should be noted that the PII values in Table 2 would be suppressed if any of the athletes were wearing footwear that were not the Nike Vaporfly or Alphafly shoes. Nonetheless, if it is assumed that the Nike Vaporfly shoes ‘life in service’ was established since approximately 2016, it is clear that the magnitude of PII increase in the period since then would not be seen as extreme when compared to the overall magnitudes of PII recorded throughout history or from those listed by Haake [5]. However, it has been paraphrased from the Burns and Tam study [1] that banning the shoes now would protect the overall integrity of the sport and its records. For example:

“I would draw it so as to preserve the performances of the past 40 years at the cost of the last three years” [40].

However, the PII progression data suggests that perceptions such as these are incorrect. The shoes’ introduction has not obtained any greater change than the reasons attributed to any former records being broken. In addition, improvements have been recorded in both footwear and track surfaces in running events [41]. Therefore, the integrity of the competitive running environment prior to the Nike Vaporfly/Alphafly introduction was arguably inconsistent already and that the ‘level playing field’ never really existed in the first place.

### Could the Introduction of the Nike Vaporfly/Alphafly Shoes Coerce Athletes to Want to Use Them?

Coercion has been cited as an effect whereby athletes have been pressured to use performance enhancing drugs in order to remain competitive [30]. There does not appear to have been circumstances whereby an athlete has been pressured to use a form of sports equipment such as footwear. However, a coercive effect could be created when statements such as “runners wearing the new Nike Vaporfly 4% running shoe broke world records over the 100-km, marathon, half-marathon and 15-km distances” are made [2]. This infers that success in the form of being the ‘fastest ever’ is hypothetically made possible (or is only possible) when using these shoes. It is also feasible that such a decision may cause athletes to feel forced to change sponsors or to break contractual agreements if they feel that their success is dependent on such shoes and if their current footwear is not considered competitive in comparison. The only way to resolve this issue pragmatically would be to ensure that all athletes have open and equal access to such shoes. Without this, a ban could be justified on the basis of this disparity. However, coercion as an effect has been marginalised as a major issue when it was proposed that the decision to use technology or not remains an athlete’s fundamental ethical choice [42]. Likewise, Lavin argues that coercion is a factor in particular when athletes are using something that is inherently dangerous [30]. It was already proposed in this paper that such shoes have no evidence to suggest that they are any more harmful or dangerous than the use of other running shoes. With this in mind, it is conceded that barriers may exist but that such barriers are created by the athlete themselves, not the sport itself.

### Does the Use of the Nike Vaporfly/Alphafly Shoes Contribute Towards Spectator Appeal?

Whether these shoes provide an added appeal to spectators is difficult to identify. Wann proposed a scale for sports fans’ motivations [43]. This scale identified eight factors that comprised a spectators motives including: eustress, self-esteem, escape from daily life, entertainment, economic factors, aesthetics, group affiliation and family needs. From these, the attraction of Nike’s shoes could fall under the ‘aesthetics’ criterion but arguably not the others. Shoes by themselves are a relatively small visual component of a runner’s appearance and are hard to see during a distance running race due to being in a constant state of motion. An elite distance runner’s foot strike cadence is arguably hard to see clearly when it is circa 2.7 Hz [44] or when changes in running cadence are subtle enough to only vary in practise by circa 1% [32]. However, technologically based controversy in itself has been cited to stimulate interest [45]. For example, the use of prosthetic limbs in able-bodied competition by Oscar Pistorius generated a significant amount of academic study [23]. However, spectator appeal that is directly attributed to sports technology is an uncommon phenomenon and has only been reported as an effect once [13]. It is therefore argued that, due to the footwear’s proportionally small size, constant motion and that competitive running can often see groups of runners clustered together, it has a less obvious and visually distinctive form than related controversies such as Pistorius’s prosthetic limbs.

### Does the Use of the Nike Vaporfly/Alphafly Shoes Affect the Integrity of the Sport or Provide an Advantage Over the Sport Itself?

It should be questioned whether use of the Vaporfly/Alphafly shoes attacks the integrity of the sport or provides an advantage over the sport itself. The PII study earlier in this paper demonstrated that use of the shoes when considered *objectively* is not out of keeping with previous leaps in running performance and this would suggest that the integrity of running is therefore practically unchanged. However, this finding may not directly address some of the concerns that current runners have raised such as:

“when a shoe company puts multiple carbon fiber plates in a shoe with cushion between the plates it is no longer a shoe, it’s a spring, and a clear mechanical advantage to anyone not in those shoes” [46]

This comment was arguably inaccurate on account of the fact that, firstly, it has been incorrectly assumed that the record breaking shoes worn by Kipchoge had multiple carbon plates [47]. This confusion was cited as potentially stemming from patents for running shoes that Nike had lodged around the same time [47]. Secondly, a conventional running sole using rubber as a material should also be considered in essence a spring. Rubber has long been used as a means of shock absorption and for energy return in footwear [48]. In addition, whilst both the Puma Brush track shoes [11] and the Ossur prosthetic limbs [49] created increased performance when running, they both only utilised *passive* not dynamic technology. Therefore, this debate is likely not about springs per se, but it is instead about one regarding *mechanical efficiency*. The foot requires an amount of energy to be administered to it, and it will then return it as efficiently as possible [50]. A shoe being a benign, inactive entity cannot produce greater than 100% of the energy put into it, so it is therefore fundamentally *restorative* in nature. The pursuit of increased efficiency occurs in many sports. Legalised examples have included crank length and cadence in hand cycles [51] or crank *q* factor width in racing bicycles [52]. Therefore, it is challenging to determine whether technology like the Nike shoes can be outlawed as this is arguably the basis for the improvement of sports technology in general, supported by the Olympic Games own mantra of ‘stronger, higher, faster’ [53].

### Does Use of the Nike Vaporfly/Alphafly Shoes Deskill or Reskill the Sport?

A key question could be whether use of the Nike Vaporfly/Alphafly footwear has somehow reskilled or deskilled the sport. Deskilling is whereby a sport is made somehow *easier* through the introduction of technology [15]. Alternatively, re-skilling is whereby its key requirements or skillsets are somehow *changed* through the introduction of technology [13]. In the case of the Nike running shoes though, these are worn in exactly the same manner as other footwear. They utilise laces and the same visual appearance of other contemporary running shoes. Superficially speaking, they are no more or less a pair of running shoes than their contemporaries. Therefore, it is a question of whether the shoes’ functionality has somehow deskilled distance running or has unexpectedly reskilled it. It could be argued that whilst the studies have proposed that an advantage exists [36] and that this would indeed reduce the time required to complete the running distance, the actual act of obtaining a world record still requires the best possible effort to achieve it. With this in mind, the Nike shoes in no way deskill running for the athletes than it would be to merely compare any time saved between any two disparate competitive running event distances. What new innovations can do though is to make cross-generational comparisons meaningless [15]. Therefore, whilst it could be argued that the sport has not been damaged in its current guise in terms of its needs or internal goods’, running’s relative simplicity as a sport means the adoption of such changes in technology invalidate comparisons to previous historical records. However, this situation has occurred before when cinder tracks were replaced with more modern running surfaces [54] and with the creation of ‘fast pools’ in swimming. At which point, whilst it could be considered disrespectful [15], such occurrences are considered normal.

### Does the Use of the Nike Vaporfly/Alphafly Shoes Somehow Dehumanise the Sport?

The question could be posed whether the use of the Nike shoes somehow dehumanises the wearer or their appearance. Dehumanisation through technological use is a relevant criticism when considering whether sports technology is ethically viable [31]. However, the use of these shoes appears visually at face value to be consistent with other contemporary shoes. They possess a sole, an upper, a lower and laces. Therefore, it would be difficult to suggest that they dehumanise the athlete any further than current running shoes. However, a product has aspects of both form and function in their design and its needs [55]. Therefore, the viewpoint regarding the shoes appearance only concerns the aspects of form. The use of the materials and the carbon plate itself relates to the shoes’ function and is a relatively recent innovation. Therefore, the question remains whether the proposed performance enhancement of 4% would be considered a ‘natural’ jump in performance [33]. There is no baseline value upon which this supposition could easily be judged to easily answer this question. A 4% increase is arbitrary in nature and could be subjectively argued to be large or small without an obvious context or baseline upon which to compare it.

### Could Financial Cost be a Barrier to Using the Nike Vaporfly/Alphafly Shoes by Athletes?

It has been argued that sports technology can act as a barrier to increased levels of participation because athletes are deterred by the high cost of ‘competitive’ equipment [15]. The Nike Alphafly/Vaporfly shoes could be considered expensive whereby they may cost as much as 400–500% more than a pair of conventional running shoes. However, the relative disparity of wealthy to poor nations [15] makes it hard to ascertain what could be considered unacceptable. Such a requirement is potentially relative when attempting to define this. For example, the cost of a new track bicycle that has been deemed legal for the 2020 Olympic Games is £15000/$19349 [56] whereas the Nike Vaporfly only costs £240/$310 [57]. In addition, a product’s cost or ‘trickle down’ of technology can make expensive products ultimately more affordable or accessible over time anyway [58]. On this general basis, the shoes’ cost could be seen as relatively acceptable to athletes, but it cannot be known if this is acceptable within the sport of running alone without a suitable evaluation of the sports stakeholders.

### Are the Nike Vaporfly/Alphafly Shoes Inaccessible to Some Athletes?

In the case of the Nike Vaporfly/Alphafly shoes, it would be a valid argument that all athletes do not have equal access to them. Many elite athletes are sponsored [59]. As a result, they may be unable to use this specific shoe product due to having a formal contractual agreement with another brand. This same issue previously occurred with the use of full-body swimsuits manufactured by the brand Speedo [13]. In that situation, it was noted that some athletes would also be unable to use the suit due to sponsorship limitations [13]. Whilst the suits’ full-body design was ultimately banned, other brands produced their own versions of the technology eventually [60]. Therefore, it could be argued that the issue regarding access was progressively diminished and therefore is less relevant as time goes on. Conversely, in the case of the Puma Brush shoe in 1968, it was reported that other manufacturers’ inability to produce the same competitive advantage helped lead to its subsequent banning from competition [12]. Whilst it is possible that a lack of access would be created if a brand enforced intellectual property rights to prevent other manufacturers from copying the technology directly, this is not always the case. It should also be noted that some of the innovations reported in the Nike shoes are not unique to them. The stiff midsole plate is not dissimilar to the flat plate shoes design proposed by Roy and Stefanyshyn [61]. In this case, they reported a 1% reduction in metabolic rate when participants ran in shoes with a flat embedded carbon-fibre plate in the midsole when compared to control shoes. More recently, another brand has already announced their own version of the carbon plate technology [62]. At which point then, the concern regarding access or intellectual property rights to the Nike shoe is likely only a short-term limitation whilst other brands adopt similar innovations or circumnavigate any legalities such as patents. At which point, whilst it is conceded that the Nike shoe designs themselves will likely be inaccessible to some athletes, the technology itself may not be.

### Resolving the Dilemma

It is fully conceded that the adoption of pragmatism does not provide solutions to inquiry that are free from criticism nor dismiss alternative viewpoints. However, in the case of the Nike Vaporfly/Alphafly running shoes, it is necessary to provide an outcome that extends upon the admission that the shoes offer a technological advantage [1] by being able to inform the sports stakeholders whether such shoes are considered illegal or unsuitable.

In the case of the ten research questions offered in this paper, each was attempted to be resolved utilising pragmatically grounded evidence and reasoning. It was proposed that there was no evidence to suggest that the shoes are any more harmful or considered any more unnatural or dehumanising in appearance than contemporary running shoes. Likewise, it was suggested that they had not provided an unnatural progression or an unusual level of performance when taking a longer term view of competitive marathon and half marathon distance running. It was also argued that the shoes would likely not be visible enough to generate spectator appeal nor change the nature of running enough to ultimately deskill or reskill it. Whilst this paper has attempted to provide a pragmatic exploration of the claims that these running shoes are unfair, it should be conceded that such innovations in footwear are at the beginning of their optimisation, not at their end. It could be assumed that the Nike Vaporfly or Alphafly were mass-produced products. However, it is entirely feasible that the shoe designs could obtain further optimisation in their stiffness [63] or energy return in the same way that lower limb running prostheses also received [50]. As a result, a pragmatic view would suggest that some level of ongoing vigilance by the sports stakeholders will likely be required to monitor the use of running shoes technologies. With this in mind, the governing body amended its rules governing competition shoes in early 2020 “to provide greater clarity to athletes and shoe manufacturers around the world and to protect the integrity of the sport” [64]. The outcome of these changes centred mainly on three fundamental rule clarifications or additions. The first was:

“any shoe that is first introduced after 30 April 2020 may not be used in competition unless and until it has been available for purchase by any athlete on the open retail market (i.e. either in store or online) for at least four months prior to that competition. Any shoe that does not meet this requirement is deemed a prototype and may not be used in competition” [65].

This rule would, at face value, outlaw use of the Alphafly shoe that Kipchoge wore for the Ineos Project run. However, this rule could arguably also be circumnavigated by manufacturers looking to maintain an advantage to their own endorsed athletes by then offering a shoe for sale in low production numbers (thereby limiting potential supply to competitors) and/or at a cost-prohibitive price (thereby making it undesirable to other consumers). It is then conceded that this could then undermine the cost-based criterion that was discussed earlier in this paper.

The next major rule changes were:

“5.13.1 (save for where Rule 5.13.2 applies) must not contain more than one rigid plate or blade made from carbon fibre or another material with similar properties or producing similar effects, whether that plate runs the full length of the shoe or only part of the length of the shoe” [65]

“5.13.3 must have a sole with a maximum thickness of no more than 40 mm”.

These rule additions would seem to be aimed directly at several of the innovations that the Alphafly and Vaporfly contained. It limits the use of the carbon plates but it should be noted that it does not limit their material composition. Additionally, it makes the assumption that sole thickness is a definitive means of performance advantage, but this has subsequently been disputed in some cases [66]. Either way, the sole thickness limitation rule will now outlaw the Alphafly shoes that were used in Kipchoge’s Ineos Project run, but the current (and thinner soled) ‘Vaporfly 4%’ and ‘Vaporfly Next%’ models (that utilise the same carbon plate and sole principles) would remain legal.

Finally, the following rule was also created:

“5.2.2 Where World Athletics has reason to believe that a type of shoe or specific technology may not comply with the letter or spirit of the Rules, it may refer the shoe or technology for detailed examination and it may prohibit the use of such shoes or technology in competition pending examination.” [65]

This rule seems to provide the governing body some flexibility to outlaw whatever shoe they wish, despite it passing the more qualitative rules discussed earlier. The expression of ‘spirit’ has caused some issues in its application to sports technology in the past due to its subjectivity and relativity [13]. Ultimately though, these new rules seem to predominantly act as a deterrent rather than to be a watertight means of removing all opportunities for innovation in the future.

There remain some contentious factors raised by the questions in this study that likely warrant further exploration in the future. Whilst it was suggested that coercion was fundamentally a matter of athlete choice, the awareness that it could exist cannot be dismissed fully. This same concern also relates to athletes being able to obtain access to the Nike shoe technology. It was stated that many athletes will be contractually limited by the shoes they can use. However, if it is accepted that the shoes are performance enhancing, but not to a level that would be considered as an advantage over the sport (when considering the PII’s calculated in this paper), this issue is considered a short-term one. Nike’s competitors will likely eventually create their own shoe technology that is similar to the Nike’s, thereby reducing any disparity. It also cannot be dismissed that much of the criticism that has been lodged publicly against the shoes could have been made by athletes that are contractually unable to use them, thereby being biased in any opinion that they have about their design or potency.

It also could not be stated with confidence that the cost of the shoes could not be considered excessive when in comparison to existing shoe products or for running as sport. Whilst this paper provided the relative example of a racing bicycle, this does not specifically address the expectations or market within the sport of competitive distance running which could well be different.

Ultimately then, whilst seven of the research questions posed in this paper propose that there are no grounds to outlaw their design, the three factors such as access, cost and coercion arguably remain as potential issues to some extent. The philosophical question that then arises is whether one or more of these factors are outweighed by the seven that are felt to not do so or whether if even one of these is enough to consider outlawing the shoes’ technology entirely. It is proposed that issues surrounding access, cost and coercion are all factors that are short termed in nature and could therefore be seen as tolerable on that basis. Either way, this paper proposes upon balance that the evidence and arguments against these shoes design are not substantial enough to warrant their exclusion from competitive running use.

## Conclusion

A pragmatic research philosophy is utilised in conjunction with a ten-item framework to determine if the Nike Vaporfly/Alphafly running shoes should remain legal for use in distance running competition. Upon review, seven of the ten items supported the continuing use of the Nike Vaporfly/Alphafly running shoe technology in competition. The use of a performance improvement index determined that the concerns regarding the shoes’ unfairness when viewed across the sports history were unfounded. However, three items were felt to be more contentious and could still be considered problematic in the short term. It was felt that on balance, the argument was proposed that in their current guise, such footwear should continue to be used in competitive distance running but would require ongoing vigilance as such technology develops in the future.

## Data Availability

Not applicable
